# Resolution of left atrial appendage thrombi: No difference between phenprocoumon and non‐vitamin K‐dependent oral antagonists

**DOI:** 10.1002/clc.23823

**Published:** 2022-04-04

**Authors:** Katharina Biller, Benedikt Biller, Hannes Findeisen, Lars Eckardt, Horst Wedekind

**Affiliations:** ^1^ Department of Cardiology I—Coronary and Peripheral Vascular Disease, Heart Failure University Hospital Münster Germany; ^2^ Department of Cardiology II—Electrophysiology University Hospital Münster Germany; ^3^ Department of Cardiology St. Franziskus‐Hospital Münster Germany

**Keywords:** atrial fibrillation, intracardiac thrombus, LAA‐thrombus, NOAC, phenprocoumon, thrombus resolution

## Abstract

**Background:**

Atrial fibrillation is the most important risk factor for left atrial appendage (LAA) thrombi, a potentially life‐threatening condition. Thrombus resolution may prevent embolic events and allow rhythm‐control strategies, which have been shown to reduce cardiovascular complications.

**Hypothesis:**

There is no significant difference between phenprocoumon and non‐Vitamin K‐dependent oral anticoagulants (NOACs) in the resolution of LAA‐thrombi in a real‐world setting.

**Methods:**

Consecutive patients with LAA‐thrombi from June 2013 to June 2017 were included in an observational single‐center analysis. The primary endpoint was defined as the resolution of the thrombus. The observational period was 1 year. Resolutions rates in patients on phenprocoumon or NOACs were compared and the time to resolution was analyzed.

**Results:**

We identified 114 patients with LAA‐thrombi. There was no significant difference in the efficacy of resolution between phenprocoumon and NOACs (*p* = .499) at the time of first control which took place after a mean of 58 ± 42.2 (median 48) days. At first control most thrombi were dissolved (74.6%). The analysis after set‐time intervals revealed a resolution rate of 2/3 of LAA‐thrombi after 8–10 weeks in the phenprocoumon and NOAC groups. After 12 weeks a higher number of thrombi had resolved in the presence of NOAC (89.3%) whereas in the presence of phenprocoumon 68.3% had resolved (*p* = .046).

**Conclusion:**

In this large observational study NOACs were found to be potent drugs for the resolution of LAA‐thrombi. In addition, the resolution of LAA‐thrombi was found to be faster in the presence of NOAC as compared to phenprocoumon.

## 1 INTRODUCTION

Over 26 million people worldwide suffer from a stroke every year. In western countries 20% of all strokes and transient ischemic attacks (TIAs) are of cardioembolic origin.^1^, ^2^ Cardioembolic strokes are more severe than other ischemic strokes.^3^, ^4^ There has been a steady increase of cardioembolic strokes in the last few years.^5^ In patients with atrial fibrillation (AF) as the most important risk factor, a consequent anticoagulant therapy avoids 70% of all cardioembolic strokes.^5^, ^6^ Therefore, the presence of intracardiac thrombi is a potentially life‐threatening condition because of the risk of embolization. To allow rhythm control in patients with AF which may be of prognostic relevance^7^ the presence of intracardiac thrombi has to be excluded in advance.

Thus, not only prevention but also adequate therapy of existing thrombi is of utmost importance. Today non‐vitamin K‐dependent oral anticoagulants (NOACs) are favored for the prevention of intracardiac thrombi in the majority of patients with AF.^8^ However, only limited data exist for the use of NOACs in the resolution of already existing left atrial appendage (LAA) thrombi.^9^ NOACs benefit from their simpler application compared to the use of vitamin K antagonists (VKAs) and are largely independent on alimentation or co‐medication. Additionally, therapeutic levels are consistent for most patients and their use is mostly restricted by renal insufficiency. In contrast, VKAs show many interdependencies especially with the frequently used antiarrhythmic drug amiodarone and therapeutic levels can vary depending on alimentation. Still, the amount of practical experience on thrombus resolution acquired over decades may favor VKA.^10^, ^11^

This study aims at comparing the efficacy of phenprocoumon and NOACs in the resolution of LAA‐thrombi in a real‐world setting.

## 2 METHODS

This analysis included all consecutive patients diagnosed with an intracardiac thrombus from June 2013 to June 2017 in a general cardiology clinic (SFH Münster, Germany). The intent was to compare the resolving potential of NOACs and the VKA phenprocoumon on thrombi of the LAA. Patients with non‐LAA thrombi (e.g., left ventricular thrombi) were excluded. The primary endpoint was defined as the resolution of the thrombus when patients presented again for follow‐up. Informed consent was obtained from all individual participants included in the study. The datasets generated and analyzed during the current study are not publicly available, as per internal protocol, but are available from the corresponding author on reasonable request.

Persistence of the intracardiac thrombi after 1 year was defined as the secondary endpoint. Controls were made by TEE and follow‐up appointments were scheduled according to hospital capacity between 4 and 13 weeks after diagnosis. All changes in anticoagulant therapy with date and specific substance were analyzed.

This study was performed in line with the principles of the Declaration of Helsinki. Approval was granted by the Ethics Committee of **“**Ärztekammer Westfalen‐Lippe” (Nr. 2019‐641‐f‐S).

LAA thrombi (*n* = 114) were diagnosed by transesophageal echocardiography (TEE; *n* = 111), computer tomography (CT, *n* = 2), or magnetic resonance imaging (MRI, *n* = 1). The vast majority of patients presented with symptomatic AF. Some patients already presented with cardioembolic complications and were diagnosed with AF in the following diagnosis of a thromboembolic event.

### 2.1 Statistical analysis

After collecting all data, the evaluation and statistical analysis was made using SPSS Version 25 for Mac OS X (SPSS Inc.). All descriptive data were specified by absolute and relative frequency and complemented with median, arithmetic mean, and SD where necessary. The *χ* ^2^ test was used to test for independency. The Kaplan–Meier estimator was used in the context of event history analysis to identify time to resolution under therapy with different types of anticoagulation. Log‐rank tests were used to test for significance. In case of small size of groups Fisher's exact test was applied.

## 3 RESULTS

### 3.1 Baseline characteristics and comorbidities

One hundred and sixty‐three consecutive patients were included, out of those 114 were diagnosed with an intracardiac thrombus located in the left atrium (*n* = 1) or LAA (*n* = 113). The remaining patients had LV‐thrombi (*n* = 36) or thrombi of other locations (*n* = 13) and were not taken into account.

After thrombus detection, overall baseline characteristics between patients on phenprocoumon and NOAC did not differ significantly (Table 1). Groups were comparable with regard to comorbidities, with a slightly lower average CHA_2_DS_2_‐VASc Score in the NOAC group (2.68 ± 1.1, *n* = 19 vs. 3.25 ± 1.8, *n* = 48; *p* = .207).

**Table 1:** Characteristics of the patient collective with LAA‐thrombi (*n* = 114) in absolute numbers and percentage including SD were needed at the time of inclusion

Baseline characteristics	Total group (*n* = 114)	Phenprocoumon (*n* = 48)	NOAC (*n* = 19)	*p* value
Sex male, *n* (%)	65 (57.0)	30 (62.5)	12 (63.2)	0.595
Age (years), mean ± SD	76.2 (12.7)	73.4 (14.0)	72.5 (8.5)	0.327
Weight (kg), mean ± SD	87.2 (23.3)	89.4 (25.1)	92.8 (24.0)	0.618
BMI (kg/m^2^), mean ± SD	29.4 (6.6)	30.0 (7.2)	30.5 (6.6)	0.251
Comorbidities, *n* (%)				
Hypertension	94 (82.5)	37 (77.1)	14 (73.7)	0.760
Diabetes mellitus	26 (22.8)	13 (27.1)	5 (26.3)	0.603
Peripheral artery disease	18 (15.8)	12 (25.0)	0	0.012
Coronary heart disease	26 (22.8)	13 (27.1)	4 (21.1)	0.412
Myocardial infarction	10 (8.8)	7 (14.6)	1 (5.3)	0.272
Stroke	11 (9.6)	4 (8.3)	1 (5.3)	0.712
Renal insufficiency^a^	27 (23.7)	12 (25.0)	1 (5.3)	0.060
Atrial fibrillation or atrial flutter, *n* (%)				0.875
No documentation of atrial fibrillation/flutter	9 (8.0)	1 (2.1)	2 (10.5)	
Paroxysmal atrial fibrillation	26 (23.2)	11 (22.9)	1 (5.3)	
Persistent atrial fibrillation	67 (59.8)	33 (68.8)	15 (78.9)	
Permanent atrial fibrillation	8 (7.1)	2 (4.2)	0	
CHA_2_DS_2_‐VASc Score				
CHA_2_DS_2_‐VASc Score, mean ± SD, median	3.38 (1.7)	3.25 (1.8)	2.68 (1.1)	0.207
Atrial dilatation, *n* (%)				0.775
No dilatation	23 (23.2)	11 (23.4)	5 (29.4)	
Slight dilatation	29 (29.3)	13 (27.7)	4 (23.5)	
Moderate dilatation	28 (28.3)	13 (27.7)	6 (35.3)	
Severe dilatation	19 (19.2)	10 (21.3)	2 (11.8)	

*Note*: Characteristics of patients on phenprocoumon and NOAC at the time of first control. The *p *value is calculated between patients on phenprocoumon and patients on NOAC.

^a^ Impairment of kidney function was defined as GFR < 90 ml/min/1.73 m² body surface area.

### 3.2 Use of oral anticoagulants

At the time of thrombus detection, 47 patients (41.2%) were already on oral anticoagulation (28 on phenprocoumon, 9 on Rivaroxaban, 6 on Apixaban, 3 on Edoxaban, and 1 on Dabigatran). In those not on OAC, anticoagulation was started with phenprocoumon (*n* = 40) or NOAC (*n* = 7 Rivaroxaban, *n* = 8 Apixaban, *n* = 1 Dabigatran) according to the decision of the treating physician. Heparin was the OAC of the first choice in 10 patients. All of these patients were critically ill, four died within 2 weeks after diagnosis, four were lost to follow‐up, and the remaining two were discontinued on anticoagulation because of bleeding complications.

If a thrombus was detected in the presence of OAC the drugs were continued in 25 patients (53.2%), changed to phenprocoumon after previous NOAC therapy in 15 out of 19 (79.0%), or from phenprocoumon to NOAC in 6 out of 28 patients (21.4%), 3 were put on Rivaroxaban and 3 on Apixaban. One patient was changed to heparin after previous phenprocoumon therapy. Overall a steady increase in the use of NOACs during the study period was noted, starting at 6.25% in 2013 and reaching 46.2% at the end of the inclusion period in 2018.

### 3.3 AF and LAA thrombi

Most patients (91.9%) with an LAA‐thrombus had documented AF or atrial flutter at the time of diagnosis. Persistent AF was the most common overall as well as in the subgroups of patients on phenprocoumon or NOAC (68.8% and 78.9%, respectively; Table 1). Correspondingly, the majority of patients for whom all relevant data points could be acquired (*n* = 99) showed varying degrees of dilatation of the LA, only 23.2% of patients showed no dilatation according to the American Society of Echocardiography (Table 1).^12^ The mean size of LAA thrombi was 128.9 mm² (±142.5) for the 38 patients for whom all relevant data points could be acquired.

### 3.4 Major adverse events

The majority of patients (88.4%) did not suffer a stroke before being diagnosed with an intracardiac thrombus. During the course of the study, 5.3% (*n* = 6; 4 on VKA, none on NOAK) of patients suffered from a stroke and 1.8% (*n* = 2; both on VKA) from a TIA. In the group of patients who had a previous stroke (*n* = 11), two patients (18.2%; both on VKA) suffered from a recurrent stroke and one patient from a recurrent TIA after the diagnosis of intracardiac thrombi was established.

### 3.5 Time of control and resolution of thrombi

Out of the 114 LAA‐thrombi, 67 (62.3%) patients were controlled at least once and 47 patients were lost to follow‐up for several reasons, for example, death (*n* = 7), referral to a secondary hospital (*n* = 4), different follow‐up modality (e.g., CT/MRI instead of TEE; *n* = 2). Yet, the majority of patients did not present again within 1 year for their scheduled follow‐up TEE (*n* = 34).

The average time to first control TEE was 58 ± 42.2 (median 48) days or 8 weeks. Disregarding the type of oral anticoagulation administered the LAA‐thrombi were dissolved in 74.6% of cases at the time of first control. Out of the 67 patients, 48 were treated with phenprocoumon. In this group 77.1% of LAA‐thrombi were dissolved, and the average time to first control was 63 days (±48.1) or 9 weeks. The INR was considered effective in 69% of patients at this isolated point in time. The remaining 19 patients were treated with NOACs and showed a resolution rate of 73.7%. The control took place after an average time of 48 days (±18.3). There was no significant difference in the resolution of LAA thrombi at the point of first control depending on the type of anticoagulation therapy (*p* = .499). Almost all patients (15 out of 16) who had no resolution of their LAA‐thrombus at the time of first control were re‐evaluated in a second control. At this time six additional LAA‐thrombi were dissolved.

### 3.6 Time to resolution

**Figure 1:**
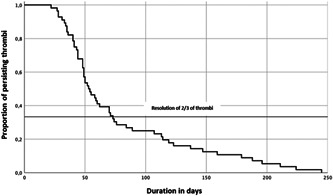
Overall time‐dependent resolution of LAA thrombi. The *x *axis shows the duration in days and the *y *axis the proportion of persisting thrombi. The value of 2/3 resolution of initially diagnosed thrombi is reached after 71 days

Out of the 67 patients who were controlled at least once, 56 showed the resolution of the thrombus within 1 year. Irrespective of the type of oral anticoagulation the average time to resolution was 77.8 (SD ± 7.4) days, the median was 53.5 days. A resolution rate of two‐thirds of thrombi was reached after 71 days (Figure 1), correspondingly a control rate of more than 80% was reached after 10 weeks (Table 2). Additionally, one patient was first controlled after 211 days and one after 245 days. Both thrombi were dissolved.

**Table 2:** Control rates and resolution of thrombi regardless of the type of oral anticoagulation

	Number of controlled patients	Number of dissolved thrombi
After 4 weeks	6/56 (10.7%)	4/56 (7.1%)
After 6 weeks	20/56 (35.7%)	14/56 (25.0%)
After 8 weeks	41/56 (73.2%)	30/56 (53.6%)
After 10 weeks	46/56 (82.1%)	36/56 (64.3%)
After 12 weeks	50/56 (89.3%)	41/56 (73.2%)

Note: Cutoffs were chosen to match realistic time frames for clinical controls. Displayed are the number of controlled patients and the number of dissolved thrombi after 4, 6, 8, 10, and 12 weeks. After 10 weeks 82.1% of patients were controlled, corresponding to 64.3% of total thrombi dissolved. Conversely out of the 46 patients controlled, 78.3% of their thrombi were dissolved. As expected, the percentage of thrombi dissolved increases with the longer time intervals.

### 3.7 Resolution of LAA‐thrombi in dependence of the type of oral anticoagulation

**Figure 2:**
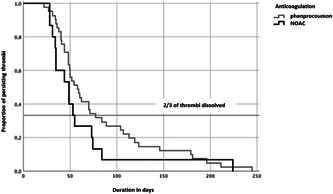
Resolution of LAA‐thrombi depending on the type of oral anticoagulation. The *x *axis shows the time in days and the *y *axis the proportion of persisting thrombi. Dark gray shows the curve when phenprocoumon was administered, black shows patients on NOACs. A resolution rate of 2/3 of thrombi is reached earlier in patients on NOACs than in patients on phenprocoumon. These data respect all changes in the anticoagulation regime, and the number of days while switching to anticoagulation was also accounted for

Both groups did not differ in their time to first control with an average of 63 and 48 days (*p* = .224) in the phenprocoumon and NOAC groups, respectively. The average time to resolution was 79.4 ± 8.6 days using VKA. In the presence of NOACs the average time was 59.7 ± 12.6 days. There is no significant difference in resolution time depending on the administered type of anticoagulation (*p* = .201) (Figure 2). The resolution of LAA‐thrombi depending on the type of oral anticoagulation is demonstrated in Table 3. Comparable percentages of controlled patients were seen, for example, after 8 weeks control rates were 82.1% and 81.3% in the phenprocoumon and NOAC group, respectively.

**Table 3:** Control rates and resolution rates under NOAC and phenprocoumon therapy

	Number of controlled thrombi	Resolution using NOAC	Resolution using phenprocoumon	*p* value
After 4 weeks	6/56 (10.7%)	2/15 (13.3%)	2/41 (4.9%)	0.234
After 6 weeks	20/56 (35.7%)	6/15 (40%)	10/41 (24.4%)	0.135
After 8 weeks	41/56 (73.2%)	11/15 (73.3%)	20/41 (48.8%)	0.066
After 10 weeks	46/56 (82.1%)	11/15 (73.3%)	26/41 (63.4%)	0.204
After 12 weeks	50/56 (89.3%)	14/15(93.3%)	28/41 (68.3%)	0.046

*Note*:  Cutoffs were chosen to match realistic time frames for clinical controls. Displayed are the number of controlled patients and the number of dissolved thrombi after 4, 6, 8, 10, and 12 weeks. Patients in both groups did not differ significantly in time to control (*χ* ^2^ = 0.367, Spearman correlation coefficient *r* = .634). Controls performed after 4, 6, 8, and 10 weeks did not differ significantly in thrombus resolution rate in the two groups, after 12 weeks a significantly higher resolution rate was found when administering NOACs.

After 4 and 6 weeks, resolution rates were overall low, but slighter higher in the NOAC group. With regard to the control after 10 weeks when overall 2/3 of thrombi was dissolved, there was a resolution rate in the VKA group of 63.4%. In the group of patients treated with NOAC, there was a resolution rate of 73.4%. After 12 weeks 90% of all patients were controlled at least once; resolutions rates were 93% in the NOAC group and 68% in the VKA group. Until the point of 10 weeks there was no significant difference in the time to resolution of LAA thrombi comparing NOACs and VKA but there is a clear trend towards faster resolution in the NOAC therapy group. However, after 12 weeks of treatment with either NOAC or VKA the thrombi had more often resolved in the presence of NAOC as compared to phenprocoumon.

## 4 DISCUSSION

Intracardiac thrombi are a potentially life‐threatening condition because of their risk of embolization. NOACs already have a significant value in the prevention of thromboembolic events in patients with AF. In this setting NOACs are the preferred treatment over the use of VKA.^8^ The major advantage for the group of NOACs is their overall better safety profile. Additional benefits include simple application with consistent and conceivable therapeutic levels for a wide range of patients with strict regulations for dose reduction and without the need for measurement of therapeutic blood levels. Moreover, NOACs profit from overall less drug interactions and their effect does not depend on alimentation.^10^, ^11^

The present study analyzed 114 patients with an LAA‐thrombus, primarily treated with NOAC or phenprocoumon. Overall, 64.4% of patients were controlled at least once. The average time to first control was 58 days or approximately 8 weeks. The therapeutic adherence of the collective in this study was as low as in comparable observational studies like CLOT‐AF.^13^ As the average time to resolution was 112 days in CLOT‐AF when administering a NOAC, in our study resolution was observed after approximately 60 days in patients with NOAC therapy. The difference may be explained by a smaller sample size in CLOT‐AF and later time to first control. In CLOT‐AF 14 of 15 patients showed resolution of the thrombus at the time of first control; one thrombus was not resolved in the run of the study.

In the NOAC group in our study 73.7% of thrombi were dissolved at the time of first control. This indicates that the first control probably took place later in CLOT‐AF compared to our study.^13^

Comparing the results of the X‐TRA trial with our data it can be noted that after 6 weeks there was a very similar resolution rate of 40% across the NOAC group as a whole. It may overall be possible that the time to first control was too short to fully investigate the dissolving potential of Rivaroxaban^9^ resulting in a resolution rate of 41.5% in X‐TRA.

Comparing the dissolving potential of NOACs with the potential of VKA reported from other studies the effectiveness of the NOACs seems to be at least as good as that of VKA. Only very limited data exist that investigates the potential of VKA in thrombus resolution. In a TEE study from Jaber et al. the resolution rate in patients, who mostly received VKA (or heparin) with a mean INR of 2.2, after diagnosis of left atrial thrombus was 80.1% after 47 ± 18 days. One may speculate that the INR levels were better controlled than in a real‐world setting.^14^ However, similar results were demonstrated in a study by Collins et al.^15^ in which the resolution rate of patients treated with VKA was 89% after 4 weeks with 18 patients included.

At the time of diagnosis about half of the patients already received therapeutic anticoagulation. Phenprocoumon was used more frequently and patients on VKAs were more likely to be continued on VKA after diagnosis of a thrombus. This fact most likely reflects the remaining uncertainness in the use of NOACs and the confidence in VKA therapy with a measurable therapeutic effect (INR) in the context of cardiac thrombus formation. In the run of the study, an increasing use of NOACs was observed as current AF guidelines prefer the use of NOACs.

After 10 weeks nearly 2/3 of LAA thrombi were dissolved. This point seems to be the best time for a first control regarding the number of tested patients and dissolved thrombi. However, comparatively low rates of resolution were found after 6 weeks, a time when one‐third of the patients had already received the first control. Scheduling the first control after 6 weeks seems a practical approach for daily clinical routine, while resolution numbers indicate this to be rather suboptimal. With regard to the resolution rates in dependence of the type of oral anticoagulation there was a trend towards earlier resolution of LAA thrombi in patients with NOAC therapy. This trend accumulated in a significant difference after 12 weeks with a benefit for NOACs (93.3% vs. 70.7%; *p* = .046). This benefit of faster resolution when using NOACs may be caused by consistent drug levels with easy dosing and precise rules for dose reduction though a selection bias in patients cannot be excluded. In addition, phenprocoumon and related substances only inhibit the synthesis of vitamin‐K‐depending coagulation factors, while Apixaban not only inactivates Factor Xa in plasma but also inactivates Factor Xa when already bound to thrombi.^16^ The clinical consequence of a potentially faster resolution when using NOACs is first and foremost reflected in the possibility for earlier controls of LAA‐thrombi making an earlier antiarrhythmic strategy for AF possible.

When deciding on the type of oral anticoagulation it should also be taken into account that the number of thromboembolic complications may be reduced by faster resolution of the thrombus.

## 5 CONCLUSION

In this large observational study the overall efficacy of thrombus resolution did not differ significantly between VKA and NOACs at the point of first TEE control. Regardless of the type of anticoagulation, 76.1% of thrombi were dissolved at first control. The cutoff value of two‐thirds of thrombi dissolved was reached faster when administering a NOAC. We found an overall favorable relation between number of controlled patients and number of dissolved thrombi after 10 weeks independent on the oral anticoagulation used. It would therefore be advisable to schedule follow‐up appointments at this time. Overall there was no difference in resolution of LAA‐thrombi when comparing NOACs and phenprocoumon in a real‐life setting. Further studies are needed to prove the role of NOACs and to differentiate between the resolving potential of thrombin‐ and Factor Xa inhibition as well as for each NOAC individually.

## ACKNOWLEDGMENT

Open Access funding enabled and organized by Projekt DEAL.

## CONFLICTS OF INTEREST

L. Eckardt reports receiving lecture honora from Boehringer Ingelheim, Daiichy Sankyo, BMS Pfizer, and Bayer Medical. H. Wedekind reports receiving lecture honora from Bayer Medical and AstraZeneca. The remaining authors declare no conflicts of interest.

## Data Availability

The datasets generated and/or analyzed during the current study are not publicly available, as per internal protocol, but are available from the corresponding author on reasonable request.

## References

[1] Krishnamurthi RV , Feigin VL , Forouzanfar MH , et al. Global and regional burden of first‐ever ischaemic and haemorrhagic stroke during 1990‐2010: findings from the Global Burden of Disease Study 2010. Lancet Glob Health. 2013;1:e259‐e281. 10.1016/S2214-109X(13)70089-5 25104492 PMC4181351

[2] Raskob GE , Angchaisuksiri P , Blanco AN , et al. Thrombosis: a major contributor to global disease burden. Arterioscler Thromb Vasc Biol. 2014;34:2363‐2371. 10.1161/ATVBAHA.114.304488 25304324

[3] Adams HP Jr , Bendixen BH , Kappelle LJ , et al. Classification of subtype of acute ischemic stroke. Definitions for use in a multicenter clinical trial. TOAST. Trial of Org 10172 in Acute Stroke Treatment. Stroke. 1993;24:35‐41. 10.1161/01.STR.24.1.35 7678184

[4] Lin HJ , Wolf PA , Kelly‐Hayes M , et al. Stroke severity in atrial fibrillation. The Framingham Study. Stroke. 1996;27:1760‐1764. 10.1161/01.STR.27.10.1760 8841325

[5] Yiin GS , Howard DP , Paul NL , et al. Age‐specific incidence, outcome, cost, and projected future burden of atrial fibrillation‐related embolic vascular events: a population‐based study. Circulation. 2014;130:1236‐1244. 10.1161/CIRCULATIONAHA.114.010942 25208551 PMC5384634

[6] Aguilar MI , Hart R , Pearce LA . Oral anticoagulants versus antiplatelet therapy for preventing stroke in patients with non‐valvular atrial fibrillation and no history of stroke or transient ischemic attacks. Cochrane Database Syst Rev. 2007 ​:CD006186. 10.1002/14651858.CD006186.pub2 PMC13591242 17636831

[7] Kirchhof P , Camm AJ , Goette A , et al. Early rhythm‐control therapy in patients with atrial fibrillation. N Engl J Med. 2020;383:1305‐1316. 10.1056/NEJMoa2019422 32865375

[8] Hindricks G , Potpara T , Dagres N , et al, ESC Scientific Document Group . ESC guidelines for the diagnosis and management of atrial fibrillation developed in collaboration with the European Association of Cardio‐Thoracic Surgery (EACTS). *Eur Heart J*. 2020. 10.1093/eurheartj/ehaa612

[9] Lip GY , Hammerstingl C , Marin F , et al. Left atrial thrombus resolution in atrial fibrillation or flutter: Results of a prospective study with rivaroxaban (X‐TRA) and a retrospective observational registry providing baseline data (CLOT‐AF). Am Heart J. 2016;178:126‐134. 10.1016/j.ahj.2016.05.007 27502860

[10] Altiok E , Marx N . Oral anticoagulation. Dtsch Arztebl Int. 2018;115:776‐783. 10.3238/arztebl.2018.0776 30602410 PMC6329367

[11] Ärzteschaft Add. Leitfaden “orale Antikoagulation bei nicht valvulärem Vorhofflimmern” Empfehlungen zum Einsatz der direkten oralen Antokoagulanzien Dabigatran (Pradaxa), Apixaban (Eliquis), Edoxaban (Lixiana) und Rivaroxaban (Xarelto). 2018. https://www.akdae.de/Arzneimitteltherapie/LF/PDF/OAKVHF.pdf

[12] Lang RM , Badano LP , Mor‐Avi V , et al. Recommendations for cardiac chamber quantification by echocardiography in adults: an update from the American Society of Echocardiography and the European Association of Cardiovascular Imaging. Eur Heart J Cardiovasc Imaging. 2015;16:233‐270. 10.1093/ehjci/jev014 25712077

[13] Fleddermann A , Eckert R , Muskala P , Hayes C , Magalski A , Main ML . Efficacy of direct acting oral anticoagulant drugs in treatment of left atrial appendage thrombus in patients with atrial fibrillation. Am J Cardiol. 2019;123:57‐62. 10.1016/j.amjcard.2018.09.026 30376957

[14] Jaber WA , Prior DL , Thamilarasan M , et al. Efficacy of anticoagulation in resolving left atrial and left atrial appendage thrombi: a transesophageal echocardiographic study. Am Heart J. 2000;140:150‐156. 10.1067/mhj.2000.106648 10874278

[15] Collins LJ , Silverman DI , Douglas PS , Manning WJ . Cardioversion of nonrheumatic atrial fibrillation. Reduced thromboembolic complications with 4 weeks of precardioversion anticoagulation are related to atrial thrombus resolution. Circulation. 1995;92:160‐163. 10.1161/01.cir.92.2.160 7600646

[16] Byon W , Garonzik S , Boyd RA , Frost CE . Apixaban: a clinical pharmacokinetic and pharmacodynamic review. Clin Pharmacokinet. 2019;58:1265‐1279. 10.1007/s40262-019-00775-z 31089975 PMC6769096

